# A Linear Temporal Increase in Thrombin Activity and Loss of Its Receptor in Mouse Brain following Ischemic Stroke

**DOI:** 10.3389/fneur.2017.00138

**Published:** 2017-04-10

**Authors:** Doron Bushi, Efrat Shavit Stein, Valery Golderman, Ekaterina Feingold, Orna Gera, Joab Chapman, David Tanne

**Affiliations:** ^1^Comprehensive Stroke Center, Department of Neurology, The J. Sagol Neuroscience Center, Chaim Sheba Medical Center, Tel HaShomer, Israel; ^2^Department of Physiology and Pharmacology, Sackler Faculty of Medicine, Tel Aviv University, Tel Aviv, Israel; ^3^Department of Physical Therapy, Sackler Faculty of Medicine, Tel Aviv University, Tel Aviv, Israel; ^4^Department of Neurology, Sackler Faculty of Medicine, Tel Aviv University, Tel Aviv, Israel; ^5^Robert and Martha Harden Chair in Mental and Neurological Diseases, Sackler Faculty of Medicine, Tel Aviv University, Tel Aviv, Israel

**Keywords:** ischemic stroke, thrombin, permanent middle cerebral artery occlusion, protease-activated receptor, endothelial protein C receptor

## Abstract

**Background:**

Brain thrombin activity is increased following acute ischemic stroke and may play a pathogenic role through the protease-activated receptor 1 (PAR1). In order to better assess these factors, we obtained a novel detailed temporal and spatial profile of thrombin activity in a mouse model of permanent middle cerebral artery occlusion (pMCAo).

**Methods:**

Thrombin activity was measured by fluorescence spectroscopy on coronal slices taken from the ipsilateral and contralateral hemispheres 2, 5, and 24 h following pMCAo (*n* = 5, 6, 5 mice, respectively). Its spatial distribution was determined by punch samples taken from the ischemic core and penumbra and further confirmed using an enzyme histochemistry technique (*n* = 4). Levels of PAR1 were determined using western blot.

**Results:**

Two hours following pMCAo, thrombin activity in the stroke core was already significantly higher than the contralateral area (11 ± 5 vs. 2 ± 1 mU/ml). At 5 and 24 h, thrombin activity continued to rise linearly (*r* = 0.998, *p* = 0.001) and to expand in the ischemic hemisphere beyond the ischemic core reaching deleterious levels of 271 ± 117 and 123 ± 14 mU/ml (mean ± SEM) in the basal ganglia and ischemic cortex, respectively. The peak elevation of thrombin activity in the ischemic core that was confirmed by fluorescence histochemistry was in good correlation with the infarcts areas. PAR1 levels in the ischemic core decreased as stroke progressed and thrombin activity increased.

**Conclusion:**

In conclusion, there is a time- and space-related increase in brain thrombin activity in acute ischemic stroke that is closely related to the progression of brain damage. These results may be useful in the development of therapeutic strategies for ischemic stroke that involve the thrombin-PAR1 pathway in order to prevent secondary thrombin related brain damage.

## Introduction

The “time is brain” paradigm is now well established in acute stroke care and emphasizes that neuronal tissue is rapidly lost as stroke progresses (1). Similarly, in permanent middle cerebral artery occlusion (pMCAo) performed in rats, infarction in the ischemic core progresses rapidly and the ischemic damage expands to the peripheral region with time (2). Understanding the mechanisms underlying the propagation of damage during stroke progression is critical for targeting effective treatments that can minimize irreversible neuronal damage.

Thrombin is a coagulation factor and a target for prevention of cardioembolic stroke (3, 4). In addition to its role in thrombogenesis, thrombin has important pleiotropic effects through the activation of its major receptor, protease-activated receptor 1 (PAR1) (5, 6). Activation of this receptor by low concentrations of thrombin may have neuroprotective effects, while at higher concentrations thrombin has deleterious effects (7–11). In the setting of an acute ischemic stroke, high thrombin levels have been detected in the infarct area, possibly as a consequence of blood–brain barrier opening (12, 13) or as a result of its local synthesis in the brain (14, 15) or both. Functionally, thrombin has been shown to cause synaptic dysfunction (15–19) and later, on vascular disruption, inflammatory response and neuronal damage (20–22), through the activation of PAR1 (13, 20). Indeed, the toxic effects of thrombin in the central nervous system have been shown in various ischemia models where neuroprotection could be achieved by PAR1 deletion (22). Recently, it was reported in pre-clinical studies that the direct thrombin inhibitor, argatroban, is protective when it is given immediately or after 1, 2, 3, but not 4 h delay from the ischemic event (23, 24). Moreover, there are clinical data that suggest that thrombin inhibition may be effective not only in prevention of cardioembolic stroke but also in the context of acute stroke (25–27). In addition, Chen et al. showed that continuous administration of PAR1 antagonist immediately after MCA occlusion decreased the level of neurovascular injury (13). However, in studies of the PAR1 antagonist, Vorapaxar (Merck, USA), in patients with prior ischemic stroke, there was an increased risk of intracranial hemorrhage without an improvement in major vascular events (28). Thus, better understanding the role of thrombin and PAR1 progression following ischemic event may have significant inputs for the development of therapeutic strategies based on thrombin-PAR1 pathway.

Thrombin also cleaves the zymogen protein C (PC) to its activated form (aPC). Effective activation of PC by thrombin requires the transmembrane glycoprotein, thrombomodulin (TM), as a cofactor. PC activation by the thrombin–TM complex is enhanced ~20-fold *in vivo* when PC is bound to the endothelial cell protein C receptor (EPCR) (29). Retention of aPC bound to EPCR allows aPC to express multiple cytoprotective activities that involve PAR1. These cytoprotective activities include aPC-mediated anti-inflammatory and anti-apoptotic activities, alterations of gene expression profiles, and protection of endothelial barrier functions (30–33). Recently, we reported that different concentrations of thrombin affect long-term potentiation (LTP) through different molecular routes converging on PAR1. High thrombin concentrations induce a slow onset LTP, whereas low concentrations of thrombin promote LTP through aPC–EPCR-mediated mechanism (18).

In order to examine the role of thrombin–PAR1–EPCR during stroke progression, we sought to determine the profiles in time and space of thrombin activity as well as the relevant PAR1 and EPCR levels in mice brains during pMCAo. Mapping thrombin activity throughout the brain provided the opportunity to assess both the ischemic core and its surrounding penumbral areas. This is of particular importance since the thrombin-PAR-1 pathway is an attractive target for the development of novel therapeutics for ischemic stroke (13, 19, 22, 23, 34–36) and may potentially prevent secondary thrombin-related brain damage. Clarifying the temporal and spatial profiles of thrombin and PAR1 following an ischemic event is fundamental for determining therapeutic strategies based on this pathway.

## Materials and Methods

### Animals

Studies were carried out on 8-week-old male C57BL6 mice (23–30 gram, Harlan Laboratories Inc., Israel). Mice were kept at the animal house in mouse cages (six/cage) with free access to food and water with a 12/12-h light–dark circle. Anesthesia was performed with 2.5% isoflurane mixed in oxygen and delivered through a facemask. This type of anesthesia is common for this stroke model. PMCAo was performed using the common filament model based on our previously reported technique (12), using silicone-coated filament (Doccol Corp, Redlands, CA, USA). Mice were sacrificed using 100 µl pental by i.p. administration. Immediately prior to sacrifice, motor deficits were measured for each animal and scored using a 5-point Neurologic Severity Scores described as follows (37): 0, no neurologic deficit; 1, failure to extend left forepaw fully; 2, circling to the left; 3, falling to the left; 4, depressed level of consciousness and failure to walk spontaneously. Mice were divided into three groups according to the sacrifice time: 2 (*n* = 5), 5 (*n* = 6), and 24 (*n* = 5) h following pMCAo. Another group of healthy mice served as control (*n* = 5). Sample sizes were chosen based on the magnitude of thrombin changes in our previous experiences (12, 38). Exclusion criteria include massive bleeding during surgery and/or intracerebral hemorrhage.

### Thrombin Activity Assay

Thrombin activity was measured using a fluorometric assay as described previously (12, 39). Briefly, thrombin activity was measured on fresh coronal slices taken from the ischemic and contralateral hemispheres by a fluorometric assay, quantifying the cleavage of the synthetic peptide substrate Boc-Asp(OBzl)-Pro-Arg-AMC (I-1560 Bachem, Switzerland). Following sacrifice, the brain of each animal was immediately removed and placed in a steel brain matrix (1 mm, Coronal; Stoelting; IL, USA). First, the brain was cut sagittally in its midline so the left contralateral and the right ischemic hemispheres were separated. Then, the brain was cut starting at its anterior side (starting at slice # 3, 2 mm anterior to the bregma), into coronal 1-mm thick slices. The slices were placed into black microplate (Nunc; Denmark) containing the substrate buffer. Measurements were performed microplate reader (Tecan; infinite 200; Switzerland) with excitation and emission filters of 360 ± 35 and 460 ± 35 nm, respectively. For calibration, known concentrations of bovine thrombin (Sigma-Aldrich, Israel) were used in the same assay.

### Distribution of Thrombin Activity Levels in Mouse Brain following pMCAo

The spatial distribution of thrombin activity in the ischemic core (coronal slice #6 as described in the previous section) was determined 24 h following pMCAo, using the thrombin activity assay and 1.5-mm diameter tissue punches that were taken from the cortical and basal ganglia areas in the ischemic and contralateral hemisphere. Each tissue sample was placed in a separate well in the microplate, and thrombin activity was measured using the thrombin activity assay. Following removal of the punch tissue samples, the punctured slices were stained using triphenyltetrazolium chloride (TTC) for infarct assessment.

### Histochemical Visualization of Thrombin Activity in Mouse Brain following pMCAo

In addition to the tissue punches technique, the spatial distribution of thrombin activity in mice brain following pMCAo was determined using a novel enzyme histochemical method for visualization of the thrombin activity location in brain slice that we have developed (38). Briefly, the method is based on the cleavage of the substrate, Boc-Asp(OBzl)-Pro-Arg-4MβNA by thrombin to liberate free 4MβNA which, in the presence of 5-nitrosalicylaldehyde (NSA), is captured, yielding an insoluble yellow fluorescent product that marks the site of thrombin activity.

Twenty-four hours following pMCAo, mice were sacrificed and their brains were immediately removed and inserted into 30% sucrose solution for 24 h at 4°C. Thereafter, the brains were cut into 20 µm coronal slices using a cryostat (Leica CM1850, Leica, Germany), and the cut sections were picked up on microscopic glass slides (Superfrost Plus, Thermo Scientific, USA). For thrombin activity staining, the slices were incubated in a solution containing 93 µl of thrombin buffer (50 mM TRIS/HCl, pH 7.0, 0.15 M NaCl, 1 mM CaCl2), 1 µl (0.6 mM) NSA, 1 µl of an aminopeptidase inhibitor bestatin (0.1 mg/ml, Cayman Chemical Company, USA) and 5 µl (0.1 mM) of the thrombin substrate Boc-Asp(OBzl)-Pro-Arg-4MβNA (GL Biochem, Shanghai, China). The stained slices were placed at room temperature for 24 h. The specificity of the method for thrombin activity was verified using sections that were incubated in the histochemical solution containing 60 µM of the highly specific thrombin inhibitor, Nα-(2-naphthylsulfonylglycyl)-4-amidino-(d,l)-phenylalanine piperidide acetate (NAPAP, Pefabloc TH, Sigma-Aldrich, Israel). The reaction was terminated by rinsing the sections in cold 50 mM TRIS/HCL, pH 7.0. Next, the sections were fixed using 4% paraformaldehyde for 20 min, washed with PBS and 0.1% Triton X-100, and incubated for 10 min with Hoechst (1:1,000, hoe-33342, Sigma-Aldrich, Israel) for nuclear staining. Finally, the sections were air dried and closed with mounting media and cover glass. Thrombin activity localization and hoechst staining were visualized using inverted fluorescence microscope (I × 81, Olympus, Japan) with a filter cube U-MWU2 (BP 300-385, BA420, DM400, Olympus, Japan).

### Western Blot

Following the thrombin activity assay, coronal slices numbers 5–7 from each hemisphere were pooled and homogenized using a pestle motor mixer (Argos Technologies, USA). Proteins from the brain homogenates were separated by polyacrylamide gel electrophoresis and transferred onto nitrocellulose membranes for western blot analysis. Membranes were incubated with primary rabbit anti PAR1 antibody (1:500, ABCAM-ab32611, Abcam, USA) and primary rabbit anti EPCR antibody (1:500, NBP2-21578, Novus, USA), followed by horseradish peroxidase-conjugated goat anti-rabbit antibody (Jackson Immunoresearch Laboratories, USA). Protein signals were visualized using enhanced chemiluminescence assay kit (Thermo Scientific, USA). Bands intensities were quantified using Image J, a java-based image processing program.

### Statistics Analysis

Statistical analyses was conducted using SPSS v. 22 for Windows (IBM, NY, USA). One or 2-way ANOVA followed by *post hoc* test was applied on normally distributed data set. For data which were not normally distributed, Wilcoxon signed-rank and Kruskal–Wallis tests were used. Pearson and Spearman correlations were used for parametric and non-parametric variables. All numerical data are expressed as mean ± SEM, unless otherwise indicated. *p*-Values of <0.05 were considered significant.

## Results

### Temporal and Spatial Profile of Thrombin Activity in Whole Slices following pMCAo

The pMCAo procedure in 11 mice resulted in consistent but moderate neurological deficits in all groups with a trend for more severe outcome in the 24 h group (median = 0, 2, 2, 3 for control mice and mice that underwent 2, 5, and 24 h pMCAo respectively; *p* = 0.001, by Kruskal–Wallis test). In order to determine the temporal profile of thrombin activity in mouse brain following a focal ischemic event, we measured brain thrombin activity levels 2, 5, and 24 h following pMCAo (Table S1 in Supplementary Material).

**Figure 1:**
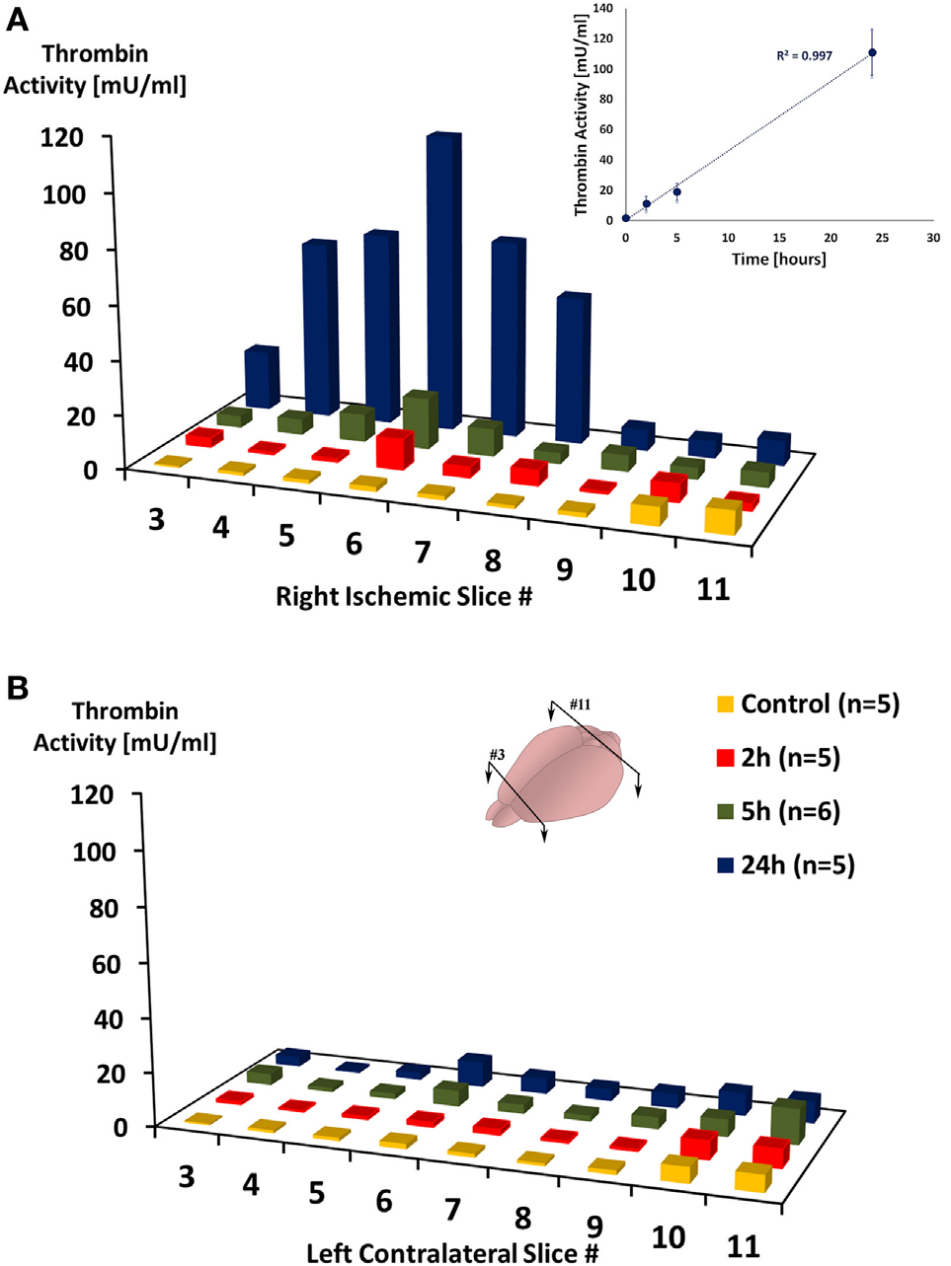
**Thrombin activity in the ischemic hemisphere following permanent middle cerebral artery occlusion**. Mean thrombin activity levels measured in brain slices taken from the right ischemic **(A)** and left contralateral **(B)** hemispheres, at the indicated time intervals after right permanent MCAo. Slices were numbered from anterior (#3) to posterior (#11), slices’ thickness = 1 mm. Inset is a plot of the mean thrombin activity levels that were measured in slice #6 as function of occlusion time. *n* represent number of mice that were used in each group.

Figure 1 presents the mean thrombin activity levels in the ischemic and contralateral hemispheres at various time intervals after pMCAo. A repeated measures model was used to assess the association between slice number, occlusion time (control, 2, 5, and 24 h), the interaction between the two, and thrombin activity. In the anterior and middle parts of the ischemic hemisphere (slices 3–8 right), a significant effect was found for slice number [*F*(1.150, 4.598) = 76.584, *p* = 0.004] and occlusion time [*F*(1.726, 6.904) = 13.826, *p* = 0.0004] with a significant interaction between the two [*F*(2.090, 8.360) = 7.271, *p* = 0.014]. In a *post hoc* analysis, the source of the difference between slice numbers was from the comparison of slices 3, 4, 5, 7, and 8 to slice 6 (*p* = 0.002, *p* = 0.016, *p* = 0.067, *p* = 0.009, *p* = 0.015). In addition, the source of the difference between occlusion times was from the comparison of control with the stroke groups (*p* = 0.047; *p* = 0.041; *p* = 0.001 for 2, 5 and 24 h, respectively). These findings reflect a substantial increase in thrombin activity at 24 h compared to 5 h post ischemia (*p* = 0.001), as clearly evident from Figure 1A. Strikingly, a linear correlation was found between thrombin activity levels that were measured in the ischemic core (slice #6) to occlusion time (*r* = 0.998, *p* = 0.001 by Pearson correlation; inset in Figure 1A). In contrast to the ischemic hemisphere, in the anterior and middle parts of the contralateral hemisphere (slices 3–8 left), although there was a moderate effect for slice number [*F*(1.888, 7.552) = 8.241, *p* = 0.013], no significant effect was found for occlusion time (*p* = 0.358) nor for the interaction between the two (*p* = 0.433, Figure 1B). At all time points, thrombin activity levels in the ischemic hemisphere were higher compared to the contralateral hemisphere {2 h [*F*(1.000, 4.000) = 21.908, *p* = 0.009]; 5 h [*F*(1.000, 5.000) = 4.934, *p* = 0.077]; 24 h [*F*(1.000, 4.000) = 42.096, *p* = 0.003]}. In the control group, no significant differences was found between the two hemispheres (*p* = 0.57). In the posterior part of the ischemic hemisphere (slices 9–11), thrombin activity was not significantly changed. No significant effect was found for either for slice number (*p* = 0.821) or for occlusion time (*p* = 0.278) in these posterior slices.

### Spatial Distribution of Thrombin Activity in Brain Slice

**Figure 2:**
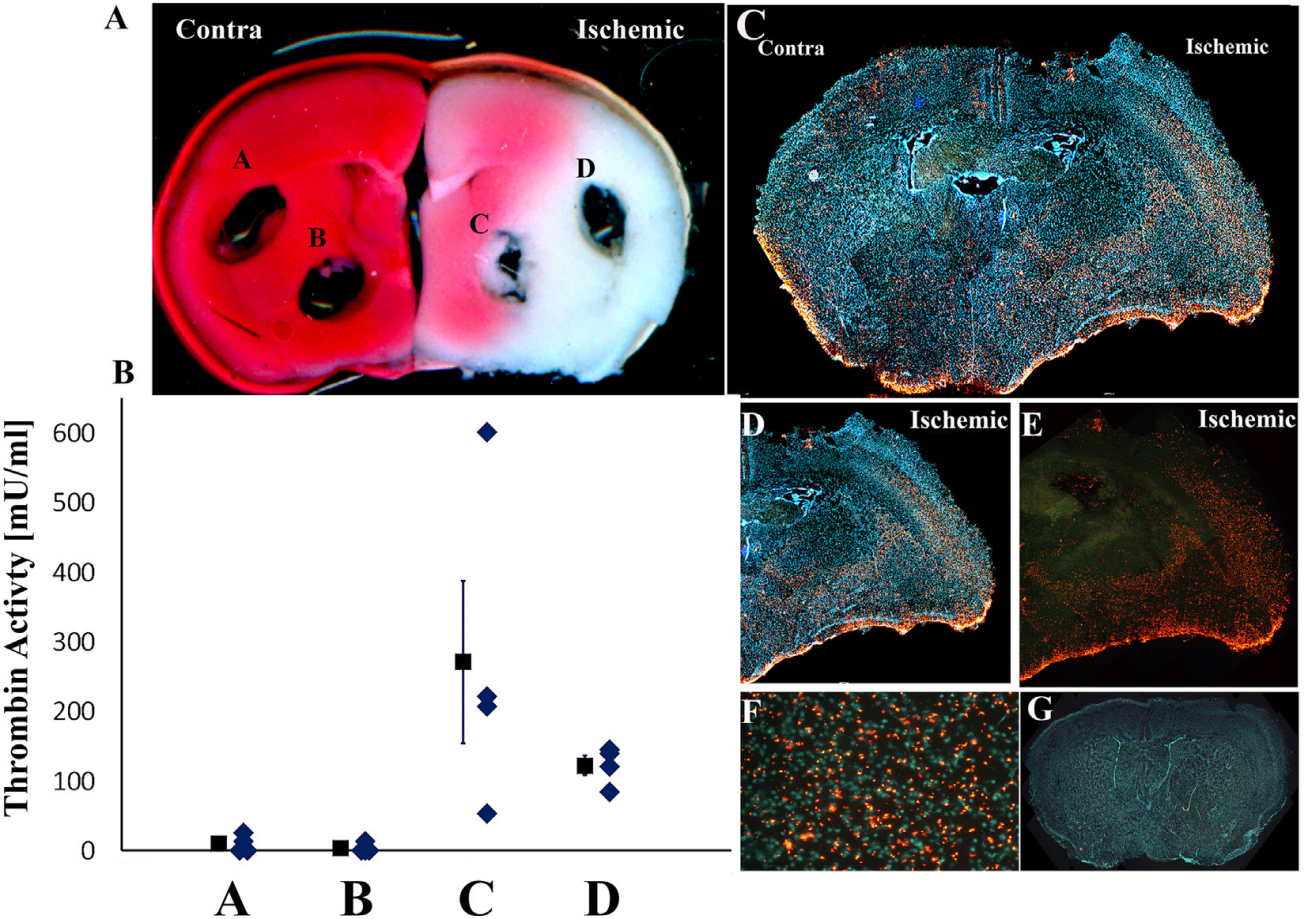
**Spatial distribution of thrombin activity in brain slice**. **(A)** The locations of the 1.5-mm diameter tissue punches sampled from fresh slices (slice location 1 mm posterior to the bregma; slice #6 in Figure 1). Typical triphenyltetrazolium chloride staining of the relevant slice used in these analyses is presented (representative of four slices developed by this method). Infarct regions are colored by white and intact brain regions by red. **(B)** Mean thrombin activity levels (milliunits per milliliter of tissue ± SEM) and its data distribution that were measured at the cortex and basal ganglia in the ischemic and contralateral hemispheres following permanent middle cerebral artery occlusion (pMCAo). **(C–E)** Fluorescence photomicrograph of coronal sections of mice brains 24 h following pMCAo that were incubated with Boc-Asp(OBzl)-Pro-Arg-4MβNA. A mosaic was formed by merging 20 pictures, each picture magnification 40× **(C)**. The small, discrete, yellow-orange fluorescent, needle shaped crystals represent the locations of thrombin activity. Cell nuclei were stained by hoechst and appear as blue spots **(C,D)**. **(F)** Higher-power photomicrograph of the typical appearance and distribution of the thrombin activity reaction product in the cortical, basal ganglia areas in the ischemic hemispheres (magnification 200×). **(G)** Absence of thrombin activity reaction product in tissue incubated in histochemical staining solution containing the specific thrombin inhibitor NAPAP. A mosaic was formed by merging 20 pictures, each picture magnification 40×.

Since thrombin levels were maximally elevated 24 h following stroke induction, we further evaluated the spatial distribution of this activity by obtaining punch samples from mice (*n* = 4) which underwent pMCAo at this time point. The localization of the standard punch samples was from the core area of the ischemic damage in the basal ganglia and in the cortical area corresponding to the penumbra (40–42). These areas (C,D, respectively) and corresponding areas in the contralateral hemisphere (A,B) are marked out in a representative slice stained for ischemic damage by TTC as presented in Figure 2A. The highest thrombin activity levels were measured in the basal ganglia of the ischemic hemisphere, and they were higher compared to those measured in the ischemic cortex (271 ± 117 vs. 122 ± 27 mU/ml; *n* = 4; *p* = 0.072, by Wilcoxon signed-rank test; Figure 2B). As expected, these thrombin activity values that were measured in both the cortex and the basal ganglia of the ischemic hemisphere were higher compared to the corresponding areas in the contralateral slices (122 ± 27, 271 ± 117, vs. 3 ± 3, 10 ± 6 mU/ml; *n* = 4; *p* = 0.039 and 0.039, respectively, by Wilcoxon signed-rank test; Figure 2B).

The distribution of thrombin activity in the core vs. surrounding areas was further evaluated using a fluorescence histochemical method. Figures 2C–E are representative slides obtained from one animal out of five examined and presents the topographic distribution of thrombin activity 24 h following pMCAo. High density of small, discrete, yellow-orange fluorescent needle shaped crystals that mark the locations of thrombin activity (Figure 2F) were observed in the right ischemic hemisphere in the cortical areas and in the basal ganglia compared to lower thrombin activity contralaterally (Figure 2C). The histochemical localization of thrombin activity corresponds to the infarct areas per TTC staining and to areas with high thrombin activity levels as determined using the thrombin activity assay and tissue punches technique (Figures 2A,B). Interestingly, higher thrombin activity levels were observed in the deep layers of the ischemic parietal cortex compared to lower thrombin activity in its upper layers (Figures 2C–E). No staining was seen in the corpus callosum of the ischemic hemisphere (Figures 2C–E) and in the sections that were incubated with the thrombin inhibitor NAPAP (Figure 2G).

### PAR1 and EPCR Levels following pMCAo

**Figure 3:**
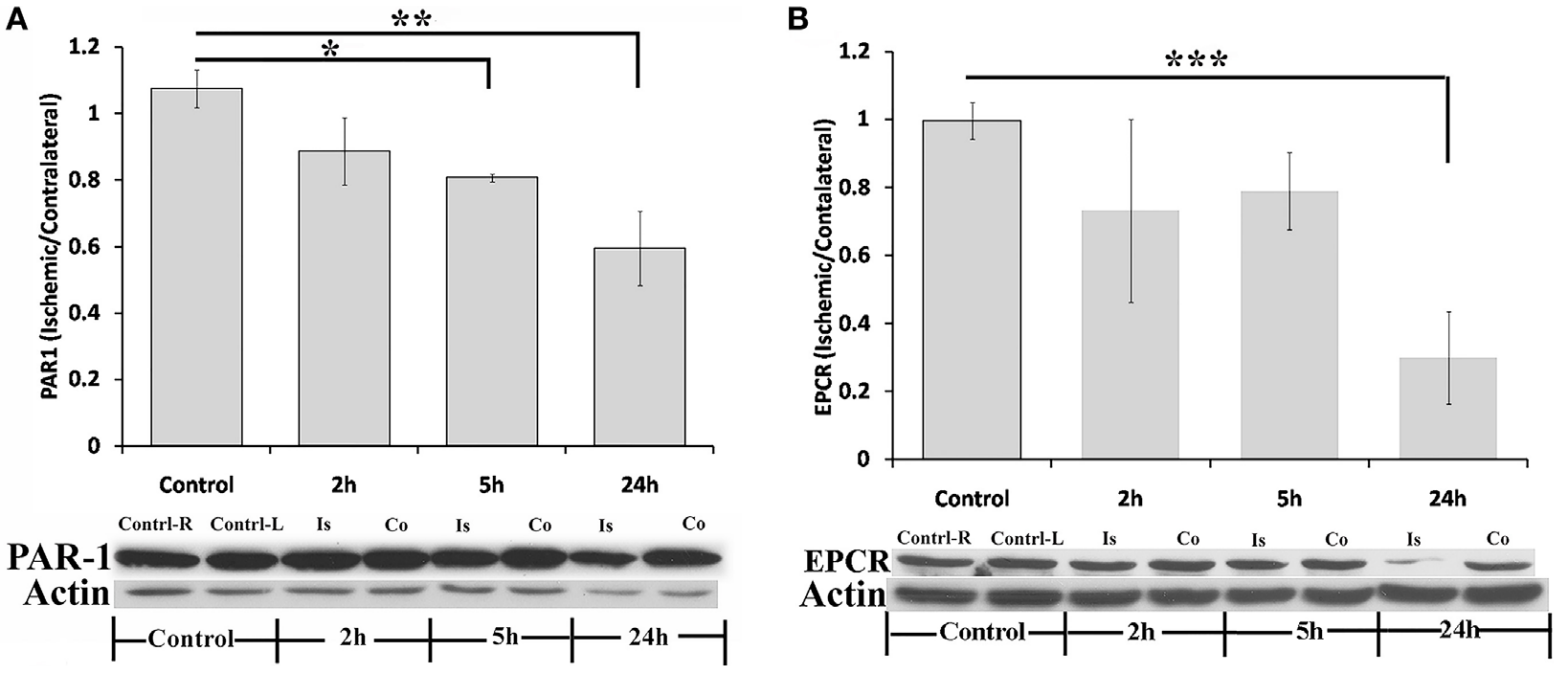
**Protease-activated receptor 1 (PAR1) and endothelial cell protein C receptor (EPCR) levels decrease following permanent middle cerebral artery occlusion (pMCAo)**. Ratio of PAR1 **(A)** and EPCR **(B)** levels in the ischemic core vs. their levels in corresponding areas in the contralateral hemisphere, as measured at various times intervals after right pMCAo (*p = 0.055, **p = 0.004, by Kruskal–Wallis test, *n* = 5; ***p = 0.047, by one-way ANOVA following *post hoc* Tukey’s test, *n* = 4;). Is = Ischemic; Co = contralateral.

**Figure 4:**
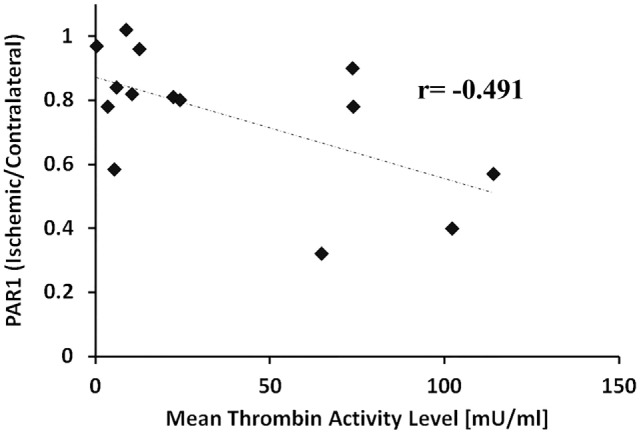
**Negative correlation between thrombin activity and protease-activated receptor 1 (PAR1) levels**. Ratio of PAR1 levels in the ischemic core vs. the corresponding areas in the contralateral hemisphere as a function of the mean thrombin activity measured in the ischemic core (slices # 5, 6, 7) 2, 5, and 24 h, following permanent middle cerebral artery occlusion. The correlation was significant by Spearman’s test (*r* = −0.491, *p* = 0.037).

Figure 3 presents levels of PAR1 and EPCR in the ischemic hemispheres relative to the contralateral hemispheres measured by western blot technique at 2, 5, and 24 h following pMCAo. In parallel to the increase in thrombin activity in the ischemic hemisphere, PAR1 levels decreased compared to the contralateral area upon MCAo. Following 5 h of pMCAo, the ratio of PAR1 levels in the ischemic core to contralateral areas was lower compared to control mice (Figure 3A, *p* = 0.055, by Kruskal–Wallis test). This decrease was further augmented following 24 h of pMCAo (*p* = 0.004; Figure 3A). There was a significant inverse correlation between thrombin activity and PAR1 level as can be seen in Figure 4 (*r* = −0.491, *p* = 0.037). Similarly, the ratio of EPCR levels in the ischemic core to the contralateral areas decreased with time after pMCAo, and this was lower compared to control mice 24 h following pMCAo (*p* = 0.047 by one-way ANOVA following *post hoc* Tukey’s test; Figure 3B).

## Discussion

In the present study, we found that thrombin activity in the ischemic core started to rise 2 h after MCAo, and activity levels continued to rise and to expand into additional areas that surround the ischemic core. Twenty-four hours after MCAo, levels of thrombin activity rose dramatically in the anterior and middle regions of the ischemic hemisphere reaching peak levels in the ischemic core. Strikingly, thrombin activity levels in the ischemic core rose linearly over time. In contrast, thrombin activity in slices that were taken from areas that are supplied by the posterior circulation (#9–11), and in the contralateral hemisphere remained as expected unchanged. Levels of thrombin activity that were measured using discrete tissue samples taken from the ischemic core reached values that are more than two times higher compared to those obtained when sampling the entire hemisphere. It is likely that the levels found when sampling the entire hemisphere are an underestimation and represent an average between higher levels in the infarct core and lower levels in perinfarct areas. The propagation of the thrombin activity across slices suggests a time-dependent expansion of the core of thrombin activity.

Previously, we reported that thrombin concentrations above 60 mU/ml are able to alter synaptic responses by increasing paired pulse facilitation as well as network excitability leading to synaptic dysfunction (39). In this study, we found a linear increase of thrombin activity and that 24 h following occlusion, thrombin activity in the ischemic hemisphere is considerably above the deleterious threshold. In contrast, thrombin activity levels that were measured in the ischemic core 5 h following MCAo were below the deleterious limit of 60 mU/ml. Interestingly, this time frame fits the therapeutic window for effective reperfusion therapy (43).

In the current study, thrombin activity levels that were measured in the ischemic core 24 h following pMCAo were higher compared to those measured in a previous study of ours (12). The reason for these differences is probably due to the different types of filament that were used to occlude the MCA in each study (commercial silicone-coated filament 4-mm length compared to heat-blunted 6–0 suture coated with poly-l-lysine). Indeed, Guan et al. showed in a MCAo model performed in rats, positive correlation between suture coating length and infarct volume (44). Likewise, our group has found a positive correlation between infarcts volume to thrombin activity levels (12, 39). Not surprisingly, thrombin activity levels that were measured in the ischemic core 24 h following 90 min tMCAo (39) were lower compared to those measured following pMCAo. It is expected that in the transient model, most of the blood vessels will undergo reperfusion in such a situation and that there will be less thrombin activity left over from the initial tissue damage as well as less propagation of thrombin activity due to tissue ischemia and continued occlusion of blood vessels. In contrast to our previous study (39), in this study, we present mapping of brain thrombin activity levels following pMCAo, at several time points from the ischemic event and not just 24 h later.

The specific time points that we used in this study represent critical stages in the progression of stroke: 2 h—acute stage, immediately after the onset; 5 h—the end of the therapeutic window; 24 h—peak point of thrombin activity following global ischemia (45) and optimal time point for TTC staining. The temporal profile of thrombin activity that is presented in this study is comparable to that found following global cerebral ischemia that was performed using bilateral common carotid artery occlusion (BCCAO) (45). Following BCCAO, brain thrombin activity slightly increased at 4 h, peaked at 24 h, and declined at 72 h. The rapid increase of thrombin activity in the first hours is compatible with the findings that thrombin activity positively correlates to infarct volume (12, 39) and to the very early appearance of ischemic lesion that was observed following pMCAo (2).

The highest thrombin activity levels measured biochemically in tissue punches were found in infarct areas based on TTC staining. This finding is in agreement with our previous finding of a positive correlation between thrombin activity level and infarct size (12, 39). Furthermore, we have found that the distribution of thrombin activity in the tested slices was similar in both the punch method and the new higher resolution histochemical method. In both methods, high thrombin activity levels were observed in the right ischemic hemisphere in the cortical areas and in the basal ganglia compared to negligible thrombin activity levels contralaterally. Interestingly, non-homogeneous distribution of thrombin activity was observed in the cortical layers of the ischemic hemisphere with higher activity in the deep layers. Furthermore, no staining of thrombin activity was seen in the corpus callosum of the ischemic hemisphere. Further experiments are needed in order to better clarify these spatial patterns.

In parallel with the linear increase of thrombin activity, PAR1 levels in the ischemic core decreased as stroke progressed. These results are in agreement with the study of Chen et al. that found decrease in PAR1 levels in rat brains in the first hours after tMCAo (13). In contrast, PAR1 levels were raised in rat hippocampal slice cultures that were subject to experimental ischemia [oxygen-glucose deprivation (OGD)] (46) and in mice brains undergoing global cerebral ischemia (45). A possible explanation for this apparent conflict is that in the MCAo model, high level of thrombin activity that originates in the blood stream (13), enters into the brain tissue through vascular disruption, and activates PAR1 on cell membranes. Consequently, PAR1 rapidly internalize and degrade. The negative correlation observed between thrombin activity in the ischemic core to the ratio of PAR1 levels in the ischemic core vs. the contralateral hemisphere emphasizes the link between PAR1 reduction and elevated thrombin activity. In contrast, in brain slices that are subjected to ischemic conditions such as OGD, no circulating coagulation factors are involved and the time points examined were somewhat shorter. Thrombin that is generated in the brain cells rises (14, 15) and as a result PAR1, its own receptor, may first increase as a complementary step, but these receptors are then cleaved and internalized leading to reduced levels. It is hypothesized that inhibiting the levels of thrombin during acute stroke will normalize PAR1 levels at the 2–24 hour time frame.

Several studies have demonstrated the neuroprotective effects of deletion, inhibition or silencing PAR1 in brain ischemia (13, 20, 47–49). In a previous study we have shown that pharmacological inhibition of either thrombin or PAR1, restores physiological synaptic plasticity that was blocked in OGD neuronal networks. Moreover, we have found that hippocampal slices prepared from PAR1-KO mice that were exposed to OGD have normal synaptic transmission (15). In the current study we found that PAR1 levels in the ischemic core start to decrease 5 h following MCAo, as a results it is likely that treatment using PAR1 inhibitors might be effective only if administered in the first hours from the ischemic event.

Endothelial cell protein C receptor decreased in the ischemic core only at a late phase, 24 h following pMCAo. Proteinase 3 (PR3) a neutrophil granule proteinase is elevated in the brain following stroke and is involved in the inflammatory process (50). Villegas-Mendez et al found high affinity interaction between the neutrophil protease PR3, and the EPCR, which results in the proteolytic degradation of the receptor (51). Degradation of EPCR with consequent loss of aPC generation is likely to contribute to the already known proinflammatory roles of PR3 (51).

The novelty of the present study is to show for the first time quantitative characterization of the temporal and spatial profiles of brain thrombin activity during the course of acute ischemic stroke. The results reveals continuous increase and spatial expansion of thrombin activity in mice brains following MCAo even in areas outside the ischemic core. Furthermore, changes in PAR1 and EPCR levels during stroke progress are presented. The potential translation of these results into the clinic is by consideration of pharmacologically inhibiting thrombin activity in the brain early after an acute ischemic event as an optional complementary therapy for acute stroke in order to prevent secondary thrombin related brain damage. However, due to the continuous increase of thrombin activity, the therapeutic dose of the thrombin inhibitor should be time dependent. In contrast to thrombin activity, the decrease of PAR1 during stroke progress suggests that PAR1 inhibition might be not affective if it will be given in time delay from the ischemic event.

The main limitation of this study is that the experiments were performed only on healthy young male mice. In order to translate the results of this study to clinical implementation, future studies should include old as well as female mice. In addition, in future experiments, it will be interesting to study the role of thrombin activity in the transient occlusion model that simulates reperfusion of the artery.

In summary, findings of this study underscore the temporal and spatial profile of brain thrombin activity during the course of acute ischemic stroke. The dramatic increase over time in brain thrombin activity is in agreement with the therapeutic window for reperfusion therapy and the known progression of brain damage and may constitute a potential complimentary target for acute ischemic stroke therapy.

## Ethics Statement

This study was carried out in accordance with the recommendations of the Institutional Animal Care and Use Committee of The Chaim Sheba Medical Center (Tel HaShomer, Israel), which adheres to the Israeli law on the use of laboratory animals and NIH rules. The protocol was approved by the Institutional Animal Care and Use Committee of Tel HaShomer.

## Author Contributions

DB designed the study, performed the experiments (thrombin activity, stroke model), analyzed data and wrote the paper. OG and EF helped with the western blot experiments and data analysis. ES and VG helped with the prepared sections for staining. JC and DT supervised the project and revised the manuscript critically for important intellectual content. All authors gave their approval to the manuscript.

## Conflict of Interest Statement

The authors declare that the research was conducted in the absence of any commercial or financial relationships that could be construed as a potential conflict of interest.

## Abbreviations

BBB, blood–brain barrier; EPCR, endothelial cell protein C receptor; NSA, 5-nitrosalicylaldehyde; OGD, oxygen-glucose deprivation; PAR1, protease-activated receptor 1; pMCAo, permanent middle cerebral artery occlusion; PR3, proteinase 3; TTC, triphenyl tetrazolium chloride.
