# Supplementary material for: A Linear Temporal Increase in Thrombin Activity and Loss of Its Receptor in Mouse Brain following Ischemic Stroke
Source: Front Neurol. 2017 Apr 10;8:138. doi: 10.3389/fneur.2017.00138 (PMC5385331; doi:10.3389/fneur.2017.00138)
Supplement: Supplementary file 1 [file Table_1.PDF]

Table I. Thrombin activity levels (mU/ml of tissue, Mean± SEM) measured in brain slices taken from the right ischemic and left contralateral hemispheres, at various times intervals (2, 5, 24 hours) after right permanent MCAo. Slices were numbered from anterior (#3) to posterior (#11), slices' thickness =1mm. No procedure was performed on control mice.

		Slice number#								
**Occlusion Time**	**Hemisphere**	**3**	**4**	**5**	**6**	**7**	**8**	**9**	**10**	**11**
Control	Right	0.7±0.2	1.3±0.3	1.4±0.5	1.9±0.3	1.6±0.2	1.3±0.1	1.7±0.5	6.9±1.6	8.8±2.3
Control	Left	0.6±0.1	0.1±0.3	0.3±0.5	2.0±0.6	0.4±0.4	1.1±0.2	1.6±0.7	6.0±1.7	6.4±2.4
2h	Ischemic	3.5±1.6	0.9±0.3	1.4±0.6	11.4±4.7	4.0±2.2	5.8±1.6	0.9±0.5	6.7±2.0	2.6±0.6
2h	Contralateral	1.2±0.6	0.5±0.5	1.1±0.7	1.9±1.3	2.1±1.3	0.8±0.5	0.9±0.5	6.9±4.4	7.0±5.0
5h	Ischemic	4.5±1.3	5.9±2.0	10.4±3.9	19.1±5.6	10.3±1.8	4.2±1.5	6.2±2.3	4.4±1.9	5.7±3.6
5h	Contralateral	4.3±1.8	1.6±1.0	2.1±1.1	6.0±2.7	3.5±1.7	2.2±1.1	4.4±1.2	6.5±1.9	13.2±8.1
24h	Ischemic	22.5±4.9	66.8±11.5	72.4±11.1	111.2±15.2	73.5±14.8	54.8±7.7	8.0±3.3	6.6±3.7	9.3±2.3
24h	Contralateral	3.7±2.3	0.8±0.5	2.7±1.8	9.6±5.3	5.9±3.2	4.5±3.0	5.5±2.7	8.5±6.0	8.3±5.1
